# Methodologies to Isolate and Purify Clinical Grade Extracellular Vesicles for Medical Applications

**DOI:** 10.3390/cells11020186

**Published:** 2022-01-06

**Authors:** Asma Akbar, Farzaneh Malekian, Neda Baghban, Sai Priyanka Kodam, Mujib Ullah

**Affiliations:** 1 Institute for Immunity and Transplantation, Stem Cell Biology and Regenerative Medicine, School of Medicine, Stanford University, Palo Alto, CA 94304, USA; asmaakbar@gmail.com (A.A.); malekianfarzane2015@gmail.com (F.M.); neda.baghban@gmail.com (N.B.); spkodam@stanford.edu (S.P.K.); 2 Department of Cancer Immunology, Genentech Inc., South San Francisco, CA 94080, USA; 3 Molecular Medicine Department of Medicine, Stanford University, Palo Alto, CA 94304, USA

**Keywords:** extracellular vesicles, stem cells, isolation and purification methods, clinical application

## Abstract

The use of extracellular vesicles (EV) in nano drug delivery has been demonstrated in many previous studies. In this study, we discuss the sources of extracellular vesicles, including plant, salivary and urinary sources which are easily available but less sought after compared with blood and tissue. Extensive research in the past decade has established that the breadth of EV applications is wide. However, the efforts on standardizing the isolation and purification methods have not brought us to a point that can match the potential of extracellular vesicles for clinical use. The standardization can open doors for many researchers and clinicians alike to experiment with the proposed clinical uses with lesser concerns regarding untraceable side effects. It can make it easier to identify the mechanism of therapeutic benefits and to track the mechanism of any unforeseen effects observed.

## 1. Introduction

Cell development and maintenance of homeostasis are related to intracellular communication, both in a specific site or between various tissues. Cells communicate via cell junctions, secretions and electrical stimuli [1,2]. Similarly, EVs are another mode of communication between cells and tissues. They carry cargos containing proteins, lipids, receptors and genetic molecules. Based on their cellular origin, different types of EVs have been identified: apoptotic bodies, micro vesicles and exosomes, to name a few [3,4,5].

Nomenclature standardization efforts have been made since the first International society for Extracellular Vesicles meeting (ISEV), but the umbrella term “extracellular vesicles” remains [6,7].

In the past couple of decades, extracellular vesicles have attracted the attention of the scientific community as a source of versatile communication mediators. Numerous studies are being performed to study them. Their characteristics, including small size, less toxicity and immunogenicity and being modifiable, make them suitable biomarkers and drug delivery vehicles [8,9,10,11].

The main challenges for studying extracellular vesicles are their isolation and characterization. There are some conventional methods including ultracentrifugation and ultrafiltration, and several novel techniques including microfluidic chips and immunoaffinity precipitation kits for this purpose [12,13,14]. The clinical use of EV can be affected by the isolation method [14,15]. Therefore, trying to choose the best protocol and customize it based on the study seems necessary.

Moreover, to study EVs, it is crucial to determine their characteristics. Various properties of EVs should be identified, such as size, density, concentration, protein and nucleotide content, surface protein and lipid structure [12,16,17]. Extracellular vesicles inherit their characteristics and content from their parent cells. Therefore, clarifying their characteristics can be used for the diagnosis and prognosis of various conditions [18,19,20,21].

In this study, sources of the extracellular vesicle are described, and various isolation and characterization techniques have been discussed.

## 2. Different Methods for EV Isolation and Purification

**Figure 1:**
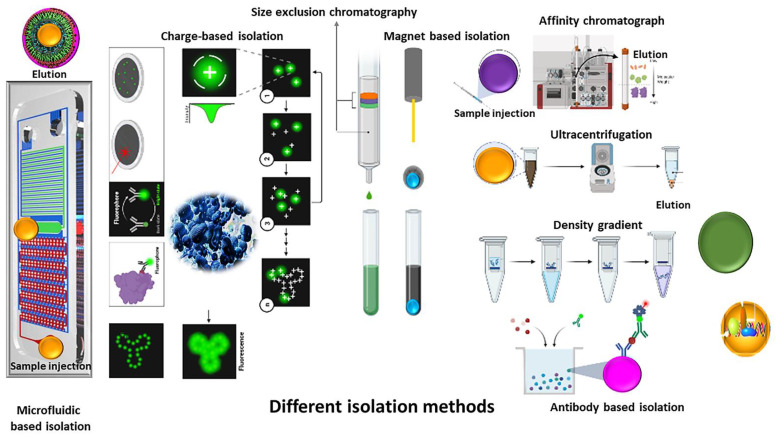
Schematic representation of different methods for extracellular isolation and purification.

In the past few decades, there has been considerable attention on using extracellular vesicles as biomarkers for various conditions and as drug delivery vehicles. One of the challenges encountered for wide application is choosing an optimum, efficient and reliable isolation method [11,22]. Filtration, ultracentrifugation and affinity separation are the most common isolation methods [23,24,25]. To isolate well-purified and healthy extracellular vesicles, a suitable combination of isolation and purification methods is necessary, Figure 1 [26,27,28].

Various peptides, proteins, lipids and cell debris contaminants are present in the source samples, some of which are similar to EVs in structure and composition, whereas some interact with EVs, preventing extraction [22,29,30].

In the following paragraphs, EV isolation methods are discussed in further detail, Figure 1.

### 2.1. Centrifugation-Ultracentrifugation-Density Gradient

A centrifuge is a device for separating particles from a solution according to their size, shape and density, and the viscosity of the medium. It causes denser elements (cells, particles, proteins) to separate at the bottom of a tube. The greater the difference in density, the faster they separate [31,32].

Ultracentrifugation is the gold standard method for extracellular vesicle isolation. The different types of ultracentrifugation are differential ultracentrifugation, density gradient centrifugation and rate-zonal centrifugation techniques [23,33].

Differential ultracentrifugation was the first technique that was used for extracellular vesicle isolation. This method is based on density, size and shape of the EVs [23,34,35,36,37,38]. The duration of centrifugation, temperature and sample dilution play pivotal role in separation efficiency [39,40]. It is easy to use and requires no or slight sample pretreatment, but it takes longer time, needs more labor and a large sample [23,41,42]. In addition, various types of EVs cannot be separated via this technique [38]. Separation via density gradient could be considered for reaching higher purification as described below [23].

Density gradient centrifugation (DGC) is another ultracentrifugation method. The difference between UC and DGC is, centrifugation occurs in a tube that contains preconstructed density gradient medium in case of DGC [23,31,35]. Sucrose and iodixanol (OptiPrep^®^) are the most used media. Through this technique, extracellular vesicles can be separated from proteins [23]. Furthermore, various kinds of EVs could be separated based on their density [14]. Longer cycle durations, low yield rate and requirement of larger volume of sample (in comparison with UC) are the drawbacks of this technique [43,44].

Gradient centrifugation is rate-zonal centrifugation based on density gradient and sedimentation rate. The sample is added to the top of the tube and through centrifugation, compounds with higher density go through the dense layer, easier that lighter compounds. The duration of centrifugation should be controlled to avoid pellet construction at the bottom of the tube [23,45]. In addition, via this technique, particles with the same density and different diameter (size) can be separated [46]. This technique causes more extracellular recovery in comparison with density gradient centrifugation [23].

### 2.2. Precipitation

This method works based on dispersibility alteration [23,31]. A water-excluding compound is first added to the sample. Polyethylene glycol (PEG), a polymer, is the commonly used compound for this purpose. After adding the polymer, centrifugation or filtration is needed for separation. The polymer dries the sample and leads to the precipitation of other molecules. [30,31,47].

Precipitation is considered a quick and easy method for EV isolation and can be used for small or large volume samples. In addition, this method requires little proficiency and not a specialized apparatus. The selectivity and quality of the isolate (unspecific co-precipitation) in this method is poor, and it must be combined with other method(s) [23,31,35,48,49]. To overcome this, filtration or ultracentrifugation can be carried out before treatment with PEG [23,31]. In addition, some commercial precipitation kits have been developed [23,50]. These kits are fast, easy to use and do not require a specific apparatus. However, they are expensive, not applicable for large samples and are not efficient to separate different types of EVs [51,52,53]. Other compounds that can be used for precipitation are Acetat salt and protamine [51,54,55,56].

Precipitation should always be followed by centrifugation and filtration to eliminate contaminants [51,54,56].

Lectin is another chemical precipitant. In this technique, the sample is pretreated via centrifugation for separating cell debris and lectin is added to the sample and incubated overnight. Lectin conjugates with the carbohydrate of the exosome membrane, changes its solubility and causes precipitation, following which exosomes/EVs will be separated via centrifugation [23,31,57]. Chemical precipitations methods are simple, cost less, and are suitable for different sample sizes [51,54,56,58,59,60].

### 2.3. Size Based Approaches

As the title suggests, EV isolation here is based on size differentiation. Various techniques purify EVs based on size, including ultrafiltration, isolation kits, sequential filtration, size-exclusion chromatography (SEC), field-flow fractionation (FFF) and hydrostatic filtration dialysis (HFD) [23].

Ultrafiltration is the most common size-based isolation method. In this technique, sample goes through membrane filters with different pore sizes. EVs are then separated based on size and molecular weight [23,31]. One of the limitations of ultrafiltration is EV clogging and trapping in the membrane. This can be prevented by initial filtration using large pore filters, followed by filtration through small pore filters [23,48,61]. The other drawbacks of ultrafiltration are poor efficiency and filter plugging [23,48,62,63]. Ultrafiltration also leads to deformation of EVs due to the pre membrane pressure (this disadvantage can be reduced by forcing lower pressure). However, the technique is still popular as it is less time-consuming and does not require expensive instruments [23,48,50,63].

In recent times, isolation kits based on size differentiation have been developed. One of them is the ExoMir Kit (Bioo Scientific; Austin, TX, USA) that contains two different membranes (upper membrane: 200 nm and downer: 20 nm) in a syringe [23].

In addition, ExoTIC (exosome total isolation chip) technology is the other kit that could purify EVs by passing the sample through different filters. These kits are easy to use, have a high yield rate and can be used for different types of bio-fluid samples [64,65]. Other methods are tangential flow filtration (TFF), direct filtration, and cyclic TFF [66].

Sequential filtration is another technique where the sample is passed through different filters. In each step particles with larger size than membrane pores are trapped, and smaller particles go through it. It is a semi-automated technique. Therefore, it is easy to use and less time-consuming [62]. Filter trapping is a limitation of sequential filtration that leads to membrane plugging and yield rate reduction [62,67,68].

Size-exclusion chromatography is another method that isolates EVs based on size. It consists of a column that allows penetration of smaller particles. This causes bigger particles to exit the column earlier. This protects the structure, integrity and biological function of EVs [23,62,69,70,71,72]. In this method, the sample does not rely on extensive pretreatment [62,70].

The first time that this method was developed, starch and water were used to form pores in the column, but through time, various other compounds such as dextran polymer (Sephadex), agarose (Sepharose) and polyacrylamide (Sephacryl or BioGel) have been used [23,62,73,74,75].

The other innovation for EV isolation based on the size differentiation is field-flow fractionation (FFF). In this method, sample is injected into a chamber that is affected via a cross flow, whereas bigger particles will be pushed to the walls of the chamber due to the cross flow, smaller particles elute earlier [23,76]. This method provides an opportunity to isolate various types of EVs and even very tiny compounds. It is faster, highly efficient, label-free and has higher sample recovery [77].

Another technique called hydrostatic dialysis isolation (HDI) uses hydrostatic forces for isolation. Small particles diffuse through the membrane and larger ones stay in the tube [23,78]. Via this method, the Tamm–Horsfall Protein, one of the abundant proteins in urinary EVs, is eliminated. After HDI, centrifugation is performed for further purification [79].

### 2.4. Affinity

Affinity based EV isolation is based on the antigens present on the EV surface. Antigens on the EV membrane are considered as markers to distinguish their sources [23,80,81,82]. These antigens are captured via specific antibodies [23,35,83]. This method provides highly purified EVs, but the harvest rate is low [23,49]. Pretreatment of samples, especially plasma, with ultracentrifugation or ultrafiltration is necessary [23,81]. In the study conducted by Tauro BJ et al., the efficacy and the results of three different methods including ultracentrifugation, density gradient isolation and immunoaffinity capture method indicate that immunoaffinity causes the highest purification of EV [80]. The limitation of this method is related to the availability of antibodies of the identified antigen. Masking of the antigens on the EV surface can prevent isolation via immunoaffinity capture methods [14]. Enzyme-linked immunosorbent assay (ELISA) is the most common immunoaffinity-based isolation and identification method [23,84]. Samples should be pretreated with ultracentrifugation before affinity capture [23,31].

One of the most effective methods to elevate EV harvest through immunoaffinity is increasing the surface area of presenting antibodies. Magneto-immunoprecipitation is a technique used for this. In this, a biotinylated antibody specific to the presenting antigen is attached to the surface of magnetic beads coated with streptavidin. Isolated EVs are then detached and used for other purposes while preserving the activity of EV protein [23,31,85]. This technique is easy and fast, but a high affinity between antigen and antibody can prevent the detachment of EVs [86].

In a study that was performed by Zhang J et.al, a combination of three methods was used to reach the optimum level of EV purification. Their protocol contains tangential flow filtration, centrifugation and immunomagnetic affinity technique; the first and second steps produce purified EVs and with immunomagnetic affinity; EVs that contain specific markers are isolated [87,88].

### 2.5. Micro-, Nano-Fluidics, Chips

Micro-, nano-fluidic chips isolate EVs based on their biochemical properties using acoustic, electrophoretic, and electromagnetic technology. This method is fast, inexpensive, efficient and can be used on small samples [23,31,89,90,91].

Microchips have been developed to isolate EVs with different approaches, including immunoaffinity, size and density-based separations. Through immunoaffinity capture, markers on the EV membrane bind to their specific antibody on the beads or inner surfaces modified by antibodies. The major limitation is the need for appearance of specific antigen on the EV surface. Developing size based microchips can surpass this limitation [91].

For size-based isolation of microchips, pressure and electrophoresis techniques are used. Electrophoresis is preferred in comparison with pressure as it prevents pore blockage [90,91].

In addition, nanowires, nano-sized deterministic lateral displacement (nano-DLD) and viscoelastic flow are the other techniques that can be used. The mechanism using nanowires is similar to SEC and contains micro-porous silicon. Nano-DLD is a pillar-array-based microfluidic method that categorizes elements in an incessant stream [91], whereas viscoelastic flow is a novel passive and label-free technique in this category that separate particles based on variance among elastic lift forces executed on compounds with different sizes in a viscoelastic medium [91,92].

Acoustic separation is one of the techniques that is used via micro-fluidic chips. In this technique, the sample is exposed to ultrasonic waves. The larger particles are affected via heavier radiation and transferred to the pressure node faster. The ultrasonic wave frequencies are controlled to separate specific particles based on the size range [23,89]. Furthermore, development of this technique produces highly purified EVs and can separate them from very low density lipoproteins with remarkable efficiency [93].

The other technique in this group is immuno-based microfluidic isolation. The mechanism is similar to ELISA. Compared with ELISA, smaller samples can be used in this method (microliter). The specificity of the method is related to the specificity of antibodies that are immobilized on the chip [23,94,95]. As mentioned before, antibodies can be loaded on the beads located on the inner surface of the microchannel [96]. To reach the mentioned specificity, Exochip has been developed recently and due to the anti-CD68 antibodies (conjugate with CD68 that is expressed on the exosomes are released via various cell types) that are fixed on the microfluidic chips, the specific EV are isolated [23,97]. ExoSearch is the other microfluidic chip for EV isolation that can be used for smaller samples and consumes lesser time [23,94]. The modified magnetic beads via specific antibodies identify CA-125, EpCAM and CD24 on the EVs of ovarian cancer [94,98].

## 3. Comparison of Different Methodological Isolation Procedures

The optimal isolation method is one of the greatest challenges for the clinical use of EVs. As mentioned before, various types of isolation methods have been developed. Each method had its own advantages and disadvantages. When considering different methods, “an ideal method for isolation of EVs should be relatively simple, inexpensive, should not require a complex or expensive equipment, should be relatively fast and allow for isolation of EVs from a large volume of samples” [31,35].

The pros and cons of each method have been described in summary in Table 1.

**Figure 2:**
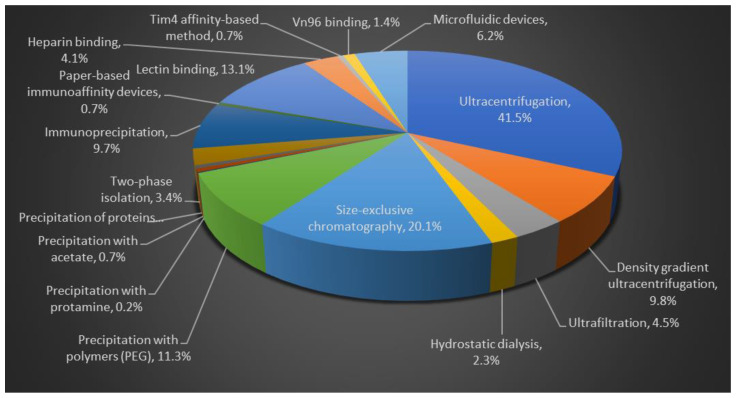
Comparison of different isolation methods for EVs purification. Flow chart data is based on published literature.

The percentages of research studies published describing each isolation method are described in pie chart, Figure 2. The comparison studies have stated that UC is the primary isolation method for 41.5% of the evaluations (for source volumes from <1 to >100 mL) [99,100].

## 4. Clinical Application of EVs

### 4.1. EVs as Diagnosis Biomarkers

Extracellular vesicles are sacs that are secreted by almost all types of cells and are responsible for intracellular communication. They inherit their content and characteristics from their donor cells [10,112,113]. Pathological and physiological characteristics of donor cells are reflected in the appearance of specific nucleotide and proteins (on the EV surface or in their content) [10,114]. In addition, the rate of EV secretion can be changed in various conditions. Therefore, EVs are precise markers for the diagnosis, prognosis and monitoring of different pathologies [113,115].

Extracellular vesicles are desirable markers for Alzheimer’s disease (AD). For instance, increased levels of Aβ1-42, total tau, p-T181 tau and p-S396 tau have been determined in the exosomes that are isolated from the plasma of AD patients compared with healthy candidates [116,117]. Additionally, lysosomal protein such as cathepsin D and LAMP1 and synaptic proteins such as synaptophysin, synaptopodin, synaptotagmin-2 and neurogranin are increased and decreased in the exosome content of plasma in AD [118,119,120]. miRNA are the other molecules that can be searched in AD patient exosomes. miR-29b, miR-181c, miR-15b, miR-146a and miR-107 are examples of miRNA that increased in AD [120].

Cancers can also be diagnosed via EV markers. Increasing in the level of some miRNAs (let-7a, miR-1,229, miR-1,246, miR-150, miR-21, miR-223, and miR-23a) in exosomes have been found in early stage of colon cancer that can be considered as biomarkers [121,122,123]. In addition, high presentation of miRNA141 and miRNA195 have been detected in circulating exosomes of early stage breast cancer patients [124]. lncRNA content of exosomes can be the other candidate for cancer diagnosis, for instance lincRNA-p21 in the urine exosomes of prostate cancer patients [20,125,126]. The main challenge for considering a specific marker on the EV is overlapping markers of different conditions. For example, upregulation of CD95L on macro vesicles have been found to be related to oral squamous cell carcinoma; however, its elevated expression is also identified in pregnancy [114,127,128]. Changes that occurred in several protein expression including psoriasin, kertain-14, galectin-7, epidermal fatty acid binding protein (E-FABP), migration inhibitor factor-related protein (MRP8) and 14 and stratifin can be considered as bladder cancer biomarkers [129,130].

Extracellular can be used for cardiovascular disease diagnosis. miRNA-133a, miRNA-143/145, miRNA-150, miRNA-155, miRNA-214, miRNA-223, and miRNA320b in exosomes are considered as markers of arthrosclerosis (AS) [131,132]. Several proteins are suggested as biomarkers of AS, including integrins derived from exosomes secreted via macrophage foam cell [133], PSMA6 (prostate-specific membrane antigen), PSMA7, and annexin A2 in blood exosomes originated from innate immune system cells [21,134] and VCAM-1 (vascular cell adhesion molecule 1) and eNOS (endothelial constitutive nitric oxide synthase) identified in plasma endothelial cell-derived exosome [131,135].

miRNA-1 and miRNA-133a alter in circulation of Acute Coronary Syndromes (ACS) and Myocardial Infarction (MI) patients [136,137,138]. Extracellular vesicle miRNA content such as miRNA-133b, miRNA-208b, and miRNA-499 are considered as MI biomarkers [138,139]. In addition, miRNA-233 is the other exosomal miRNA that can be used for diagnosis of ischemic stroke [138,140]. The other examples of exosomal miRNA with diagnostic potential are miRNA-34a, miRNA-146a, MiRNA-92, miRNA-192, and miRNA-194 are associated with heart failure [138,141,142,143].

Furthermore, proteins of exosomes can be changed in cardiovascular disease that can be considered as biomarkers. TNF-a increases in hypoxia condition [144,145], angiotensin II (AngII) type 1 receptor is overexpressed in high pressure [145], polygenic immunoglobulin receptor (pIgR), complement factor C5a (C5a) and cystatin C are upregulated in Acute Coronary syndrome [146] and Complement C1q subcomponent subunit A (C1QA) and Complement C5 (C5), Apolipoprotein D (APOD) and Apolipoprotein C-III (APOCC3) and Platelet glycoprotein Ib alpha chain (GP1BA) and platelet basic protein (PPBP) are associated with MI [138,144,147].

Moreover, extracellular vesicles can be used for diagnosis and prognosis of some infectious diseases. Some examples are as follows: Akt and CD9 in exosomes increase during urinary tract infection and can be identified as biomarkers [148]. Exosomal lncRNA-HEIH increases in Chronic hepatitis C (CHC) related hepatocellular carcinoma and can be introduced as a marker [149]. Upregulation of neurofilament-light (NF-L), high mobility group box 1 (HMGB1) and amyloid β in circulating exosomes of HIV-infected patients can indicate neuronal damage via this virus [150,151].

Therefore, extracellular vesicles are potential biomarkers of various conditions. Their content and their release can be changed via a variety of diseases. to use EVs as biomarkers, optimal isolation methods and standardized characterization protocols are necessary for this purpose [120].

### 4.2. EVs as Therapeutic Vehicle

In addition to being markers, EVs are identified as biological vehicles. They can carry various kinds of compounds including peptides, proteins, lipids, nucleic acids (RNA, DNA) and carbohydrates. In addition, extracellular vesicles, especially exosomes, can be modified and loaded with various cargoes [152,153,154]. There are different types of extracellular vesicle loading methods that provide appropriate situation to load and deliver different compounds, these methods include, incubation, transfection, physical treatment (sonication, extrusion, freeze–thaw treatment, electroporation and surfactant treatment and dialysis), in situ association and synthesis [152,155].

Currently, the potential of different kinds of nucleic acids (including miRNA, siRNA, lncRNA, mRNA, DNA, etc.) against various conditions has been cleared [156,157,158]. Their clinical application is faced with various challenges such as degradability in blood that cause short half-life, immunogenicity, accumulation in kidney and live and disability to cross hydrophilic membrane [159,160,161,162,163,164]. Therefore, it seems crucial to develop an appropriate and efficient delivery vehicle [157]. A wide variety of studies have been performed using exosomes as delivery vehicle for nucleic acids. Among various types of EVs, micro vesicles are the most appropriate vehicle for them [165]. The summary of studies that deliver miRNA and siRNA via exosomes have been described in Table 2 and Table 3.

The evaluation of effects of exosomes derived from embryonic stem cells reveals that exosomes can transfer their mRNA content to the target cells and protect their functional activities [194,195]. In addition, exosomes derived from mesenchymal stem cells contain IGF-1 m-RNA that causes expression of IGF-1 and leads to renoprotective effects [196].

In addition, some studies have evaluated EVs to deliver peptides and proteins. For instance, exosomes loaded with Survivin-T34A cause growth limitation of prostate tumors via apoptosis induction [197]. Catalase, a redox enzyme, was loaded in macrophage-derived exosomes and injected intravenously to the mouse model of acute brain injury that lead to neuroprotective effects [198]. In the other study, human MUC1 (hMUC1) has been expressed in two MHC type-distinct mouse cell lines. The result of the study indicate that the engineered exosomes can stimulate immune system and suppress hMUC1-expressing tumor growth, specifically and efficiently [199]. In the other study, performed by Admyre C, a combination of 23 immunogenic peptides from EBV, CMV and influenza virus were loaded into monocyte-derived DC exosomes and exhibited immune system induction via stimulation of CD8+ T cell [200].

Furthermore, extracellular vesicles can carry some other compounds including synthetic and natural molecules. Doxorubicin [201,202], paclitaxel [203] and rhodamine 123 [204] delivery to the cancer cells and dopamine transferring into the brain [205] are the examples of the synthetic molecules. Curcumin and celasterol are the compounds that, when divided from plant extracts, have also been loaded into exosomes and used against inflammation and cancer, respectively [57,206,207,208].

Overall, it seems that extracellular vesicles are successful in embracing different types of cargoes. Furthermore, Extracellular vesicles can be modified for target therapy. Different approaches have been investigated for this purpose that was mentioned before [209,210]. The strategy for EV modification is related to the condition or characteristics of target cells [155]. The most common modification approach is designing exosomes via specific ligands to bind to target receptors. For this aim, there are two different techniques: 1. Direct assembling, 2. Transfection. Her2 and EGFR are located using these techniques and targeted in breast cancer and colorectal cancer, respectively [155,211,212]. The other approach for EV engineering is using pH-sensitive peptides on their surface, which in acidic environments causes deformation releasing the cargo [155,213]. In addition, magnetic directing can be used to direct therapy towards the target. EVs that are designed by magnetic compounds are directed by outer magnetic force to the target area [155,214]. This revealed the potential of EVs to load various cargoes and then modify as an appropriate vehicle for different molecules [155].

## 5. Conclusions

Isolation and characterization of EVs needs more attention to reach a high efficacy and specificity. For this, the characteristics of sample and study should be considered. In addition, advantages and disadvantages of methods as well as their properties and conditions help us to choose the most effective method [215,216]. Standardization of purification methods would be a great step to understand the requirements. Clear classification of these methods based on the characteristics of EVs, prognostic, diagnostic and clinical requirements, cost-effectiveness would be prudent. The main areas to focus on with regard to isolation and purification are high selectivity and greater efficiency [114,217,218]. This will increase the reach of EVs for clinicians and scientists to try the EVs as the need arises. This in turn would increase the knowledge regarding their behavior in clinically relevant scenarios.

## Figures and Tables

**Table 1:** Comparison of EV isolation techniques in terms of source, recovery, purity, sample volume and time.

Method	Sources	Time	Volume	Recovery	Purity	Ref
Ultracentrifugation	MCF-7 cell line	4 h	1 mL	-	Moderate	[44]
Ultracentrifugation	Non-Small-Cell Lung Cancer (NSCLC) SK-MES-1 cell line	20 h	500 µL	70%	<UF	[63]
Ultracentrifugation	Human colon carcinoma LIM1863 cells	2h	500 μL	5–25%	Low	[80]
OptiPrep™ density gradient centrifugation	Human colon carcinoma LIM1863 cells	>21 h	500 μL	5–25%	>UC	[80]
OptiPrep™ density gradient centrifugation	human breast cancer cell line MCF-7	20 h	1 mL	-	Very high	[44]
Density Gradient centrifugation	Tca8113 human tongue squamous cell carcinoma cell line	20 h	>1 mL	>UC	Similar to UC	[101]
ExoQuick-TC™ precipitation	human breast cancer cell line MCF-7	13 h	1 mL	-	Low	[44]
ExoChip	Blood serum	<2 h	<400 μL	Low	-	[97]
TEI precipitation	human breast cancer cell line MCF-7	13 h	1 mL	-	Low	[44]
Ultrafiltration	Non-Small-Cell Lung Cancer (NSCLC) SK-MES-1 cell line	18 h	500 µL	90%	>UC	[63]
Sequential filtration	MDA231 breast cancer cells	-	150 mL	<UC	High	[102]
heparin/polymer-coated microspheres	Plasma	1 h	2 mL	81%	High	[103]
Heat Shock Protein (HSP)-binding peptide Vn96	HT-29 cell	32 min	2 mL	Poor	Poor	[104]
Liquid biopsy chip + HSP-binding peptide Vn96	MCF7	20 min	0.2 mL	99%	-	[105]
Enzyme-linked immunosorbent assay	LNCaP cell line HCT116 cell line	2 h	100 μL	75–80%	-	[34]
Integrated microfluidic platform	Plasma	2 h	30 μL	>99.9%	-	[106]
anti-EpCAM coated magnetic beads	Human colon carcinoma LIM1863 cells	Overnight	>1 mL	5–25%	>UC	[80]
Acoustic Nanofilter	Red blood cells	<30 min	50 μL	>80%	-	[89]
Microfluidic ExoSearch chip	Blood	>40 min	20 μL	42–97.3%	-	[94]
Immune-microfluidic	Cell line (ovarian cancer C30)	~100 min	30 μL	>99.9%	-	[95]
Microfluidic affinity separation chip	Serum	20–40 min	20–100 μL	~60%	-	[98]
Micro fluidic viscoelastic flows	Serum	<5 min	<100 μL	>80%	>90%	[92]
Microfluidic viscoelastic flow	Blood	∼25 min	-	> 99%	∼98.4%	[107]
Double-filtration microfluidic device	Urine	<10 min	<100 μL	74.2%	-	[108]
Modified acoustic	Blood	25 min	100 μL	82%	98%	[109]
Crossflow microfiltration	Lipo246 cell line	30 min	-	32–76%	Low	[110]
Centrifugal microfluidic	Human breast adenocarcinoma cell line, MCF-7 Lung adenocarcinoma cell line, H1975	<4 min	<10 μL	90%	85%	[111]

**Table 2:** Therapy based on miRNA-loaded exosomes.

Cargo	Donor Cell	Target Cell	Condition	Loading Method	Isolation Method	Route of Administration	Result	Ref
Cancer Therapy								
Let-7a	HEK293 cell expressing GE11	EGFR-expressing breast cancer	Breast cancer	Pre-transfection	ultracentrifugation	i.v	Tumor growth inhibition	[166]
miR146-b	MSC	Glioma	Primery brain tumor	transfection using electroporation	ExoQuick-TC	intratumoral	Tumor growth inhibition	[167]
miR-143	Human bone-marrow-derived	Osteosarcoma cell line 143B	Osteosarcoma	Transfection using lipofectamin	ultracentrifugation	Not applicable	Migration inhibition	[168]
miR-122	MSC	Hepatocellular carcinoma cells	Hepatocellular carcinoma	Transfection by plasmid	ExoQuick-TC	intratumoral	Enhancing chemotherapeutic sensitivity, tumor growth inhibition	[169]
miR-134	Hs578T and Hs578Ts(i)_8_	Hs578T and Hs578Ts(i)_8_	Breast cancer	Transfection	ultracentrifugation and ExoQuick,	Not applicable	Reducing migration and invasion, Enhancing chemotherapeutic sensitivity	[170]
Anti-miR-9	MSC	Drug resistant glioblastoma multiforme (BT145and BT164)	Brain tumor	Pre-overexpression	Ultracentrifugation and Total Exosome Isolation kit from Invitrogen	Not applicable	Enhancing chemotherapeutic sensitivity	[171]
Neurodegenerative diseases								
miR-219	Dendritic cells	oligodendrocytes	multiple sclerosis and dysmyelinating syndromes	-	ExoQuick and precipitation with centrifugation	Intra nasal	increase baseline myelination, reduce oxidative stress, and improve remyelination	[172]
miR-17-92	MSC	Cerebral cells	post–middle cerebral artery occlusion (strock)	Transfection with a miR-17–92 cluster plasmid	multistep centrifugation	intravenous	improvement of neurological function and oligodendrogenesis, neurogenesis, and neuritis	[173]
miR-133b	MSC	Cerebral cells	middle cerebral artery occlusion (MCAO) (stroke)	infected with lentivirus constructed with the vectors of LentimiRaGFP-hsa-miR-133b Vector	multistep centrifugation	intra arterially	enhancing neurological recovery and plasticity post stroke	[174]
miR-124	Bone marrow from adult male mice	Ischemic cells	stroke	electroporation	ultracentrifugation	intravenous injection	promoted cortical neural progenitors to obtain neuronal identity and protect against ischemic injury	[175]
miR-30d-5p	adiposederived stem cells (ADSCs)	Cerebral cells	stroke	Transfection by Lipofectamine^®^ 2000	ultracentrifugation	injection through the tail vein	suppressing the inflammatory response and preventing cerebral injury by inhibiting outophagy-mediated microglia polarization to M1	[176]
Cardiovascular diseases								
miR-19a, miR-451	mesenchymal stem cells (MSC) overexpressing GATA-4	neonatal rat ventricles cardiomyocytes	MI	Naturally exist in exosomes of donor cells	Precipitation (CBI)	intramyocardial injection	reduced apoptosis of cardyomyioyte and enhanced resistance to cardyomyocyte hypoxia	[177,178]
miR-146a	human CDCs (or normal human dermal fibroblasts [NHDFs]	Neonatal rat cardiomyoctes (NRCMs)	MI	Naturally exist in exosomes of donor cells	Exoquick ExosomePrecipitation Solution (System Biosciences)	intramyocardial injection	redevelop injured heart muscle	[177,179]
miR-21	cardiac progenitor cell (CPC)	mouse cardiac endothelial cells	MI	Naturally exist in exosomes of CPC	precipitated with ExoQuick TC (System Biosciences)	In vitro	inhibiting role in the apoptosis pathway via downregulating programmed cell death 4	[177,180]

**Table 3:** Summary of studies conducted on siRNA as a cargo of exosomes.

Cargo	Donor Cell	Isolation Method	Loading Method	Condition	Biological Effect	Ref
PLK1 siRNA	human embryonic kidney (HEK) cell	ultracentrifugation	electroporation	Bladder cancer	Effective delivery of PLK1 siRNA	[181]
GAPDH siRNA	Engeneered self-dendritic cells to express Lamp2b	ultracentrifugation	electroporation	Alzheimer’s disease	knockdown of BACE1	[182]
Alexa flour 488 labeled siRNA	HeLa and ascites with the presence of exosomal marker proteins HLA-ABC and CD63 on the membrane of these exosomes by dot blot analysis.	ultracentrifugation	chemical treatment (lipofectamin)	cancer	Silencing of RAB51	[183,184]
siRNA	NIH3T3 cells	ultracentrifugation	electroporation	lymphoma	Silencing of c-Myc and stimulation of caspase-3	[184,185]
siRNA	HEK293T cell (transduced by a lentiviral vector bearing-LAMP2b-DARPin G3 chimeric gene)	sequential centrifugation	electroporation	HER2-positive breast cancer (SKBR3 cells)	Down regulating the TPD52 gene	[186]
BCR-ABL siRNA	HEK293T (transfected with of IL3-Lamp2b plasmid DNA)	ultracentrifugation	Transfection with Lipofectamin	Chronic Myeloid Leukemia	inhibit Bcr-Abl and cancer cell growth	[187]
siRNA or shRNA targeting KRAS	normal fibroblast-like mesenchymal cells	ultracentrifugation	electroporation	Pancreatic cancer	suppressed cancer in multiple mouse models	[188]
siRNA	human induced pluripotent stem cells (huiPSCs)	centrifugation	electroporation	Pulmonary inflammation	Efficient delivery of the target siRNA into HMVECs, inhibiting the ICAM-1 protein expression	[189]
opioid receptor mu (MOR) siRNA	human embryonic kidney 293T cotransfected with siRNA and the RVG-Lamp2b plasmid using Lipofectamine 2000)	using an exosome isolation kit (Invitrogen)	transfection	morphine addiction	reduces MOR mRNA and protein levels in Neuro2A cells and the mouse brain	[190]
hydrophobically modified small interfering RNAs(hsiRNAs)	U87 glioblastoma cells	ultracentrifugation	co-incubation	Huntington’s disease	bilateral silencing of Huntingtin mRNA	[191]
SiRNA	embryonic cortical neuronal culture	ExoQuick (based on the production company’s instruction)	electroporation	Spinal cord-injury	knockdown of ASC protein transformation and significant decrease in caspase 1 activation	[192]
siRNA	Human hepatoma cells HuH7	ultracentrifugation	Tranfection (Lentiviral vectors LV-shCD81 and LV-shNS5b were constructed. LV-shNS5b contains expression cassettes of shRNA and targets the viral NS5b region)	Hepatitis C virus infection and other liver disease	suppression of CD81 expression in target hepatocytes	[193]

## Data Availability

Not applicable.
